# Sucrose Phosphorylase and Related Enzymes in Glycoside Hydrolase Family 13: Discovery, Application and Engineering

**DOI:** 10.3390/ijms21072526

**Published:** 2020-04-05

**Authors:** Jorick Franceus, Tom Desmet

**Affiliations:** Centre for Synthetic Biology (CSB), Department of Biotechnology, Ghent University, Coupure Links 653, 9000 Ghent, Belgium; jorick.franceus@ugent.be

**Keywords:** glycoside phosphorylase, glycosylation, sucrose, glucosylglycerol, glucosylglycerate, carbohydrates, GH13_18

## Abstract

Sucrose phosphorylases are carbohydrate-active enzymes with outstanding potential for the biocatalytic conversion of common table sugar into products with attractive properties. They belong to the glycoside hydrolase family GH13, where they are found in subfamily 18. In bacteria, these enzymes catalyse the phosphorolysis of sucrose to yield α-glucose 1-phosphate and fructose. However, sucrose phosphorylases can also be applied as versatile transglucosylases for the synthesis of valuable glycosides and sugars because their broad promiscuity allows them to transfer the glucosyl group of sucrose to a diverse collection of compounds other than phosphate. Numerous process and enzyme engineering studies have expanded the range of possible applications of sucrose phosphorylases ever further. Moreover, it has recently been discovered that family GH13 also contains a few novel phosphorylases that are specialised in the phosphorolysis of sucrose 6^F^-phosphate, glucosylglycerol or glucosylglycerate. In this review, we provide an overview of the progress that has been made in our understanding and exploitation of sucrose phosphorylases and related enzymes over the past ten years.

## 1. Introduction

High sugar consumption is associated with several adverse health effects such as obesity, metabolic syndrome and dental caries [1,2]. Therefore, the World Health Organisation strongly recommends that the dietary intake of sugars should be reduced, causing governments around the world to increase health awareness and take legislative measures such as the introduction of taxes on sugar-sweetened beverages [3,4]. These trends are slowing down the growth of demand in the global sugar market, while changes in market regulation (e.g., the end of sugar production quota in the EU) are resulting in oversupply and falling prices. To combat these challenges, there is a need for innovative alternative uses of surplus sucrose. An attractive solution is offered by transglycosylation technologies that can convert sucrose into a wide range of valuable products. Examples of such products are rare sugars with health-promoting properties or glycosides of small molecules like vitamins, flavours, antibiotics and fragrances.

**Figure 1:**
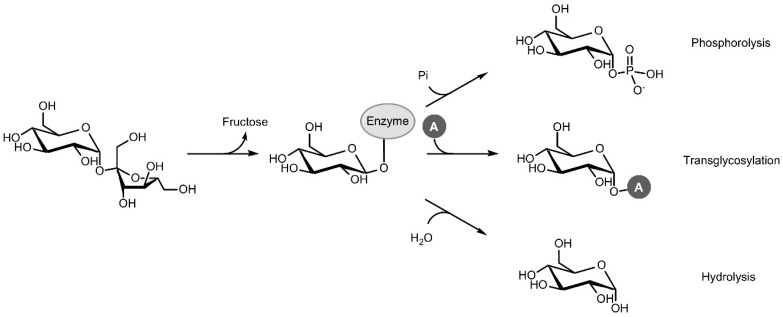
Phosphorolysis, transglycosylation and hydrolysis reactions catalysed by sucrose phosphorylase (Pi: inorganic phosphate, A: acceptor).

Sucrose phosphorylase (SP; EC 2.4.1.7; sucrose:phosphate α-d-glucosyltransferase) is a promising enzyme in this respect. In vivo, SP catalyses the reversible phosphorolysis of sucrose with inorganic phosphate, producing α-d-glucose 1-phosphate (Glc1P) and fructose. The reaction proceeds through a double displacement mechanism where a carboxylic residue attacks the anomeric carbon of sucrose, resulting in a covalent β-glucosyl-enzyme intermediate that can be intercepted by phosphate in the next step (Figure 1) [5]. In vitro, however, SP can be applied as a versatile and efficient transglycosylase for two major reasons. The first is its renowned acceptor promiscuity, which allows the glucosyl-enzyme intermediate to be intercepted by a diverse group of compounds other than phosphate as well, effectively transferring the glucosyl moiety of sucrose to those compounds. The second is the fact that sucrose is a very powerful donor with a reactivity that rivals that of activated donors such as UDP-glucose [6]. High transglycosylation yields can thus be reached. However, the double displacement mechanism also comes with one important downside. The glucosyl-enzyme intermediate can be intercepted by water too, resulting in irreversible hydrolysis of the donor substrate. Hence, tilting the ratio of transglycosylation over hydrolysis through process or enzyme engineering is usually imperative to achieve satisfactory results.

The last comprehensive review on SP was published in 2010, when Goedl et al. discussed the enzyme’s basic biochemical and structural properties and provided an excellent overview of its first applications as a transglucosylation catalyst [7]. Since then, the collection of interesting reactions that can be performed by SP has only expanded, and the enzyme has been the subject of several engineering studies with the aim of broadening or improving its industrial potential even further. Moreover, significant advances have since been made to our understanding of the family that SP belongs to. Despite being regarded as a transferase in the EC classification system, SP is most closely related to the glycoside hydrolases in family GH13 of the Carbohydrate-Active Enzyme database (CAZy; http://www.cazy.org), where it is found in subfamily 18 (GH13_18). Until a few years ago, GH13_18 was thought to contain only sucrose phosphorylases, but some of the family members were recently found to act on sucrose 6^F^-phosphate, glucosylglycerol or glucosylglycerate instead. In this review, we focus on the work that has been done on SP and its related enzymes in GH13_18 in the past decade.

## 2. Discovery of Novel Glycoside Phosphorylases in GH13_18

### 2.1. Sucrose 6^F^-Phosphate Phosphorylase

**Figure 2:**
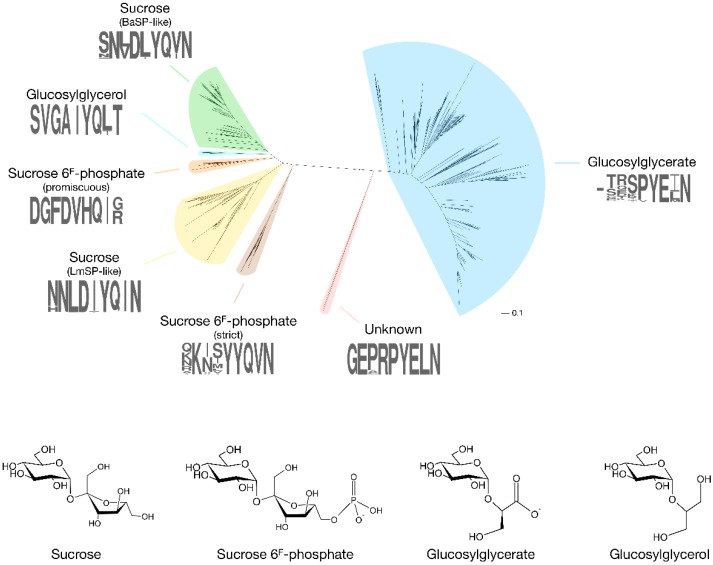
Phylogenetic tree of GH13_18 proteins with the specificities that have been discovered so far (BaSP: *Bifidobacterium adolescentis* SP; LmSP: *Leuconostoc mesenteroides* SP). A sequence logo of the loop A region (positions 339–347 in BaSP), which acts as a specificity fingerprint, is shown for each clade. The tree was obtained by extracting all sequences in subfamily GH13_18 from the CAZy database, followed by alignment with ClustalO and tree construction with PhyML 3.1 using default parameters [13,14].

The first enzyme in GH13_18 that does not prefer sucrose as a glucosyl donor was found rather coincidentally [8]. In search of an SP that is more thermostable than the ones isolated from mesophilic sources such as *Leuconostoc mesenteroides* or *Bifidobacterium adolescentis*, Verhaeghe et al. looked through the CAZy database for sequences of putative SPs from thermophilic organisms. Although several sequences originated from species with a high optimal growth temperature, only those from the order *Thermoanaerobacterales* were located in a clade with proven SP activity in the subfamily’s phylogenetic tree. A representative protein from *Thermoanaerobacterium thermosaccharolyticum* showed significant activity on sucrose, and with an optimal temperature of 55 °C and a melting temperature at 79 °C, it was definitely the most thermostable SP ever reported. However, its affinity for sucrose (*K*_M_ of 76 mM) was remarkably low compared to other SPs (*K*_M_ of 1–15 mM). It was eventually discovered that the enzyme has a clear preference for sucrose 6^F^-phosphate (*K*_M_ of 13 mM). The enzyme phosphorolyses this phosphorylated sugar into Glc1P and fructose 6-phosphate, explaining the presence of genes encoding a putative phosphofructokinase and a phosphoenolpyruvate-dependent transport system in the same operon. It is possible to differentiate these sucrose 6^F^-phosphate phosphorylases (SPP; EC 2.4.1.329) from SPs by looking at the sequence stretches that correspond to the loop regions in subsite +1. The differences are most obvious in loop A (residues 339–347 in *B. adolescentis* SP), situated between strand β7 and helix α7 of the catalytic (β/α)_8_-barrel domain that is typical of the GH13 family [9]. In this loop, the N(L/V)D(I/L/V)YQ motif that is indicative of SP activity is replaced by a GFDVHQ motif in TtSPP (Figure 2, Figure S1).

More recently, phosphorylases from *Ruminococcus gnavus* E1 and *Ilumatobacter coccineus* were also found to possess SPP activity [10,11]. Unlike TtSPP, which is very promiscuous and shows high activity on sucrose, these two SPPs are strictly specific to sucrose 6^F^-phosphate. They are also clustered in a different, but close clade of the subfamily’s phylogenetic tree, and they are characterised by a KXXYYQ motif in loop A. Clearly, evolution seems to have found two acceptor site architectures that both successfully establish high SPP activity. This is also apparent from the crystal structures of *I. coccineus* SPP (IcSPP; PDB code: 6S9U) and TtSPP (PDB codes: 6S9V). Their monomers show a strong structural similarity to *B. adolescentis* SP (BaSP; PDB codes: 1R7A, 2GDU, 2GDV), sharing a (β/α)_8_-barrel and a C-terminal domain made up of antiparallel β-sheets (PDB codes: 6S9V for TtSPP, 6S9U for IcSPP, 1R7A/2GDU/2GDV for BaSP) [9,12]. However, domains B (between strand β3 and helix α3) and B’ (between strand β7 and helix α7), which shape the acceptor site, are much more variable between the three enzymes.

Genomic environment analysis highlighted a real relationship between SPP activity and the presence of a kinase domain in the same gene cluster [10], strengthening the hypothesis that SPP is involved in an unconventional metabolic pathway for sucrose. Sucrose can either be phosphorylated upon translocation by a phosphotransferase system, or sucrose 6^F^-phosphate originating from a currently unknown environmental or metabolic source can be taken up as such. Afterwards, it is broken down by SPP to form Glc1P and fructose 6-phosphate. The latter can then be converted to fructose 1,6-diphosphate by phosphofructokinase.

### 2.2. 2-O-Glucosylglycerate Phosphorylase

By expressing a few enzymes from a very large unexplored clade of the phylogenetic tree, we noticed that the putative SPs from *Meiothermus silvanus*, *Spirochaeta thermophila* and *Escherichia coli* are unable to catalyse the phosphorolysis of sucrose. Instead, they are strict 2-*O*-glucosylglycerate phosphorylases (GGaP; EC 2.4.1.352) [15,16]. Their true specificity was derived from their genomic organisation, where they are frequently accompanied by genes encoding a glycerate kinase, glucosyl 3-phosphoglycerate synthase or glucosyl 3-phosphoglycerate phosphatase. Glucosylglycerate is a compatible solute that can be accumulated in large amounts to protect the cell against osmotic stress while remaining compatible with cellular functions [17], and at this time, GGaP is the only known possible metabolic sink for glucosylglycerate in many of the organisms that synthesise it. Although the enzyme’s exact metabolic function has not yet been experimentally confirmed, it might act as a regulator of the intracellular levels of the glucoside. Curiously, plenty of organisms that are not believed to accumulate glucosylglycerate also possess a gene that encodes a putative GGaP. An in-depth investigation of the in vivo purpose of the phosphorylase in various organisms would be helpful to solve some of the remaining uncertainties surrounding glucosylglycerate and its metabolic pathways.

Like SPPs, GGaP can easily be distinguished from SPs based on sequence data alone. Most obvious is the (T/S)ETN motif at the tip of β-sheet 5 of the (β/α)_8_-barrel (Figure S1). A second example is their conserved Glu residue in loop A, which is replaced by Gln in most other clades (Figure 2).

### 2.3. 2-O-Glucosylglycerol Phosphorylase

Another novel phosphorylase specificity was discovered by searching GH13_18 for sequences that do not contain the characteristic motif of SP, SPP and GGaP in loop A. Sequences with a peculiar VGAIYQ motif were found in a clade that is interlocked between those of SPs and promiscuous SPPs (Figure 2, Figure S1). Expression and characterisation of an enzyme from *Marinobacter adhaerens* from this clade revealed that they are strict 2-*O*-glucosylglycerol phosphorylases (GGoP; EC 2.4.1.359) [18]. Glucosylglycerol is a compatible solute, just like glucosylglycerate, and GGoP is the only known enzyme that can catalyse its phosphorolysis in glucosylglycerol-producing organisms. The enzyme might be involved in a catabolic pathway to salvage the glucoside when the environmental conditions no longer require the presence of intracellular osmolytes, but this hypothesis has yet to be verified.

It is worth noting that glucosylglycerol phosphorylases also exist in family GH65 of the CAZy database [19]. However, those invert the anomeric configuration upon phosphorolysis, yielding β-glucose 1-phosphate instead.

### 2.4. Mysterious Myxobacterial Phosphorylases

An isolated clade in the phylogenetic tree of subfamily GH13_18 contains a few enzymes from *Corallococcus* species that do not contain the signature sequence patterns of the characterised phosphorylases, with a GEXRPYE motif in loop A and an AETD motif in the loop that holds the catalytic acid/base residue (Figure 2, Figure S1) [20]. Despite an extensive screening of possible substrates, their function remains obscure at this time. *Corallococci* and other myxobacteria are a prolific source of secondary metabolites with unusual biological activities that support their complex life cycle and social behaviour [21], so the metabolic purpose of the enigmatic phosphorylase is definitely worth investigating further.

## 3. Application of Sucrose Phosphorylase and Related Enzymes

### 3.1. Synthesis of Phosphorylated Sugars

The most obvious application of GH13_18 phosphorylases is the exploitation of their native phosphorolytic function to produce Glc1P. Glc1P is the preferred glycosyl donor for performing transglycosylation reactions with phosphorylases found in families GH13, GH94, GT4 and GT35, but it can also be an effective donor in the chemical synthesis of glycosides [22,23,24]. Other possible applications of Glc1P include its use as a nutritional supplement to stimulate intestinal active calcium transport, or as a substitute for inorganic phosphate in parenteral nutrition [25,26]. A production process for Glc1P involving GH13_18 phosphorylases would only be economically viable when starting from sucrose with SP. Glc1P is a reactive compound, with a free energy of hydrolysis of −5.0 kcal/mol at 25 °C [27]. However, sucrose is even more reactive than Glc1P (−6.6 kcal/mol), meaning that high yields can be achieved when this cheap disaccharide is used as the starting material [6].

Generating Glc1P from sucrose with SP is quite straightforward, and a few high-yielding continuous processes have already been described. One made use of the thermostable BaSP, which was immobilised on SepaBeads EC-HFA to enhance its kinetic stability even further, allowing a packed-bed reactor to be operated at 60 °C for at least two weeks without any loss of activity [28]. In a different example, multiple silica-binding modules (Z_basic2_) were fused to the SP from *Leuconostoc mesenteroides* (LmSP) to attach the enzyme to the wall surface of glass microchannels in a microstructured reactor [29]. Excellent space–time yields of ~200 g/L/h could be achieved with both processes, although their performance varied heavily depending on the chosen process parameters.

**Figure 3:**
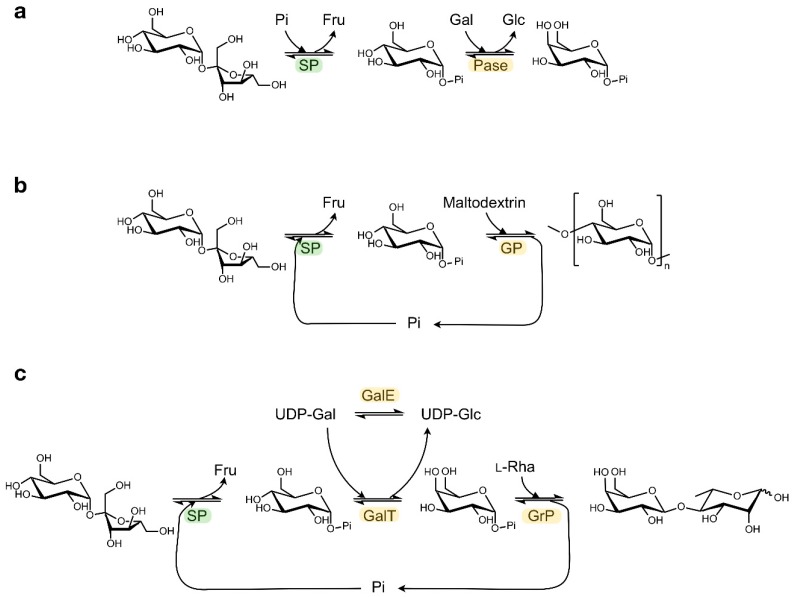
Examples of cascade reactions with SP. Synthesis of (**a**) α-galactose 1-phosphate, (**b**) amylose, (**c**) β(1,4)-Galactosyl l-rhamnose. (Pi: phosphate; Fru: fructose; Glc: glucose; Gal: galactose; Rha: rhamnose; Pase: α-glucose 1-phosphatase; GP: α-glucan phosphorylase; GalT: UDP-glucose—hexose 1-phosphate uridylyltransferase; GalE: UDP-glucose 4-epimerase; GrP: β(1,4)-Galactosyl l-rhamnose phosphorylase).

Other sugar 1-phosphates beyond Glc1P can be generated by coupling SP with a promiscuous α-glucose 1-phosphatase that can catalyse transphosphorylation reactions (Figure 3a) [30]. In this way, the phosphate group from Glc1P is transferred to other monosaccharides such as mannose, galactose or N-acetylglucosamine with absolute axial selectivity in yields of up to 70%. This strategy complements the more complex kinase-catalysed syntheses of glycosyl phosphates which require expensive NTP phosphoryl donors that have to be regenerated in situ [31].

In addition, a diverse array of valuable non-phosphorylated end products can be obtained by coupling the SP activity to other enzymes that can further process Glc1P in a one-pot cascade reaction (Table 1) [32]. In the most straightforward set-up, the released Glc1P is immediately employed as glycosyl donor by configuration-inverting β-glucoside phosphorylases or by configuration-retaining α-glucoside phosphorylases, as described for the production of synthetic amylose or short-chain cellodextrins (Figure 3b) [33,34]. Alternatively, Glc1P can first be converted into a different glycosyl phosphate by phosphomutases or isomerases, thereby unlocking the subsequent activity of phosphorylases that prefer other donor substrates. The synthesis of rare sugars like β(1,4)-galactosyl l-rhamnose from sucrose can be enabled in that way (Figure 3c) [35,36,37]. Obviously, other biocatalysts beyond phosphorylases can be employed downstream of SP too, as was demonstrated in the production of glucaric acid from sucrose in a seven-enzyme cascade [38]. Furthermore, the atom efficiency of certain cascade processes can be boosted by added auxiliary enzymes that convert side products back into substrates [37]. Multi-enzyme systems may seem rather complicated at first glance, but they are often simple one-pot reactions where all enzymes, substrates and cofactors are mixed together. Therefore, they do have potential to be scaled up. Back in 2007, Nishimoto et al. reported the kilogram-scale preparation of lacto-N-biose I, a building block of human milk oligosaccharides, by the concurrent action of four biocatalysts [39,40].

Finally, the natural activity of SP can also be useful in in vivo biosyntheses. A major advantage of working with living cells is their ability to conveniently regenerate expensive cofactors like ATP with their native metabolic pathways. In one study, the overexpression of LmSP allowed permeabilised *E. coli* to efficiently produce UDP-glucose from sucrose and UMP by generating a steady intracellular pool of Glc1P as intermediate, while deletion of the gene coding for phosphoglucomutase prevented this pool from being depleted via glycolysis [41].

### 3.2. Transglycosylation for the Synthesis of Glycosides and Rare Sugars

The promiscuous nature of SPs and some SPPs allows these enzymes to transfer the glucosyl group of the donor substrate not only to phosphate, but also to many other molecules. The resulting glycosylated molecules can show drastically altered physicochemical and biological properties, and they hold great potential for applications in the food, pharmaceutical or personal care industries. The advantage of an introduced sugar moiety is most evident in hydrophobic compounds, which become more soluble in water, but it can also improve the stability of labile compounds, change the taste of flavours, establish a controlled release of fragrances or drugs, and so on [42]. The power of SP as a catalyst to produce such interesting glycosides has been well documented before [7], but plenty more examples and helpful findings have surfaced over the past years.

The most famous phosphorylase-catalysed transglycosylation process is undoubtedly the synthesis of 2-*O*-α-glucosylglycerol from sucrose and glycerol by LmSP [43]. It is currently implemented on an industrial scale by the German company bitop AG, which has commercialised the product as a moisturising ingredient for cosmetics under the tradename ‘Glycoin^®^ natural’. Bolivar et al. have since developed a continuous process where a chimaera of LmSP and a Z_basic2_ silica-binding molecule was non-covalently immobilised on the microchannel walls of a microstructured flow-channel reactor [44]. It achieved similar titers and yields as production by the soluble enzyme, with the added advantage of enabling simple reuse of the enzyme. A different immobilisation strategy involved the incubation of BaSP with glucosylglycerol prior to covalent crosslinking of the enzyme [45]. This technique, named molecular imprinting, slightly modifies the three-dimensional structure of the biocatalyst by noncovalent interactions with the target compound and subsequently locks the protein in its new conformation by immobilisation. The obtained imprinted cross-linked enzyme aggregate (iCLEA) was far more thermostable than the soluble protein, and its activity was significantly improved, although the procedure to prepare iCLEAs is perhaps less convenient in an industrial setting. The most recent process optimisation was centred around the downstream processing of the product solution. A large excess of glycerol has to be added to the reaction in order to kinetically suppress the hydrolytic side reaction that is inherent to the double displacement mechanism of SP, but the surplus glycerol must be removed from the mixture eventually. By screening several membranes at varied pressure and temperature, a suitable purification process based on discontinuous diafiltration could be established to obtain a glycerol-free glucosylglycerol solution [46].

The success story of glucosylglycerol sparked interest in the SP-mediated synthesis of similar compounds. Two groups investigated whether several non-natural diols can be supplied to LmSP or BaSP as acceptors to form the corresponding glucosides [47,48]. Small diols are generally converted in useful yields, and there is typically a preference for regioselective glucosylation at the 2-position when one terminal hydroxyl group of glycerol is substituted. However, compared to reactions with glycerol, it tends to be more difficult to suppress donor hydrolysis and formation of glucobiose side products in reactions with similar acceptors. Furthermore, it is interesting that SP barely discriminates between enantiomers of glycerol-like acceptors in many cases [47], whereas it is strictly stereoselective in others [48,49]. For instance, LmSP can achieve chiral resolution of racemic glyceric acid amide through transglycosylation, forming *(R)*-2-*O*-α-glucopyranosyl glyceric acid amide as a single transfer product [49].

When acceptors are more hydrophobic, their low solubility in aqueous systems, together with the low affinity of SP towards such molecules, becomes a major hurdle, urging the addition of cosolvents like dimethyl sulfoxide or methanol. However, those solvents are often incompatible with applications of carbohydrate-derived products, and their presence gives rise to enzyme inhibition and denaturation [50]. The ionic liquid AMMOENG 101 was found to be a particularly effective alternative cosolvent in reactions with long-chain alcohols, flavonoids, alkaloids, phenolics and terpenes [51]. It proved to better dissolve these compounds while also being less deleterious for the stability of SP compared to more common organic solvents. Another interesting alternative is the use of a liquid–liquid biphasic system containing water and a water-immiscible organic solvent. By shaking or stirring, the hydrophobic acceptor that is present in the organic phase can be transferred to the aqueous phase that contains the enzyme and sucrose. Especially ethyl acetate was a very favourable organic phase in reactions with BaSP, allowing the glucosylation of several monoterpenoids, phenolics and alkyl gallates in decent yields [52].

Cosolvent systems and biphasic catalysis have been put to use in a study where a library of antioxidant glycosides was created and characterised [53]. The α-glucosides of hydroquinone, pyrogallol, catechin, quercetine and other phenolics were obtained with the help of BaSP and a mutant TtSPP, after which these were further converted into the corresponding cellobiosides and cellotriosides by a cellodextrin phosphorylase. Thanks to this effort, the properties of these compounds could be determined and compared. All evaluated phenolic glycosides were more soluble and stable than their respective aglycons. Moreover, most were still very potent antioxidants, despite their lowered radical-scavenging abilities. These properties were influenced by the anomeric configuration, point of attachment and chain length of the glycosidic group.

A different oxidant that can be glucosylated in a fully aqueous system, but only under specific reaction conditions, is l-ascorbic acid or vitamin C. It is an important ingredient in cosmetics, food and pharmaceuticals, but it is inherently unstable. In contrast, its 2-*O*-α-glucoside is known to be more stable while retaining its biological activity, because it can be slowly hydrolysed by an α-glucosidase present in human epithelial tissue [54]. SP can efficiently and selectively produce the desired glucoside in a narrow pH window (pH 4.8–6.0 with a sharp optimum at pH 5.2) [55]. The unusual pH dependence is probably caused by charge repulsion at higher pH values between l-ascorbic acid (pKa~4.2) and the catalytic residue Glu232 (pKa~5.8) that provides base assistance.

Another very interesting application of SP and SPP arises from their ability to transfer the glucosyl group of the donor to other carbohydrates, forming new sugars. In fact, under certain reaction conditions, SP will hydrolyse its donor substrate (sucrose or glucose 1-phosphate) and subsequently recognise the released glucose as acceptor substrate in a transglycosylation reaction, forming a glucobiose. The obtained reaction products have been observed to be either a mixture of the α-(1,2)-bonded kojibiose and the α-(1,3)-bonded nigerose [56], or a mixture of kojibiose and the α-(1,4)-bonded maltose [47]. Although the rare glucobiose products are valuable, they will usually be regarded as undesired side products in transglycosylation reactions with other acceptors. Apart from glucose, plenty of other monosaccharides can also behave as an acceptor. Ketohexoses were reported to be processed by LmSP with the following order of productivity: d-fructose ≈ d-allulose > l-sorbose > l-tagatose > l-fructose > d-sorbose > l-allulose ≈ d-tagatose [57].

Finally, it is important to realise that the performance of transglycosylation reactions will vary depending on which SP is used. Aerts et al. screened the SPs from *Streptococcus mutans*, *Lactobacillus acidophilus*, *B. adolescentis* and three different *L. mesenteroides* strains on eighty putative acceptors from different structural classes [58]. Activity was detected on almost all compounds with all enzymes, although unfortunately, the reaction rate was often comparable to that of the competing hydrolytic reaction (Figure 1). Nevertheless, there are a few interesting differences between the enzymes. In general, the *L. mesenteroides* NRRL B1355 SP had the broadest acceptor specificity, and it was also the only enzyme with significant activity on a few trisaccharides. However, it showed no activity on catechol, unlike the two other *L. mesenteroides* SPs. The SP from *S. mutans* had the narrowest specificity, but its exceptionally high activity on l-arabinose demonstrates that it can outcompete the other enzymes in some cases nonetheless. The latter SP also shows the highest affinity for sucrose, which may thus be correlated with its stricter specificity in the acceptor binding site. Indeed, the strict SPP from *I. coccineus* has a much lower *K*_M_ for its native acceptor fructose 6-phosphate than the promiscuous one from *T. thermosaccharolyticum* [10,11]. Furthermore, different phosphorylases can exhibit a completely different regioselectivity as well. BaSP attaches a glucosyl group to the middle hydroxyl group of pyrogallol and related molecules, whereas TtSPP glucosylates the outer hydroxyl group instead [53]. It is clear that SP-catalysed transglycosylation reactions typically require diligent finetuning of the process parameters (chosen biocatalyst, substrate concentrations, pH) in order to achieve a satisfactory result.

## 4. Engineering of Sucrose Phosphorylase and Related Enzymes

### 4.1. Thermostability

Carbohydrate conversions in industry are preferably operated at elevated temperatures to prevent microbial contamination and to avoid excessive viscosity. The most thermostable true SP known to date is BaSP, with an optimal temperature of 58 °C [59]. Unfortunately, it quickly loses activity at the industrially relevant temperature of 60 °C [50]. A combination of sequence-based and structure-based mutagenesis was applied to BaSP in pursuit of a variant with a higher kinetic stability [50]. The first strategy was focused on a cluster of three consecutive aspartate residues at positions 445–447 that form the most flexible region of the entire protein, as indicated by their B-factor. By simultaneously mutating the residues at positions 445 and 446 to residues that occur more frequently at those positions in the alignment of all GH13_18 sequences (Pro and Gly, or Pro and Thr), the enzyme’s residual activity after one day of incubation at 60 °C could be increased substantially. Interestingly, individually substituting the two aspartates had no favorable effect at all. The second strategy involved inspecting the crystal structure of BaSP to select promising mutations on a purely rational basis. Two beneficial variants (Q331E and Q460E-E485H) were designed to introduce additional salt bridges, while one (R393N) sought to alleviate a potential electrostatic repulsion at the dimer interface. Combining all mutations together, the half-life time of BaSP at 60 °C was increased dramatically from 24 h to 62 h.

A different work attempted to create a more thermostable SP through consensus engineering, a popular and straightforward approach where each amino acid is replaced by the most frequently occurring one at the same position in a multiple sequence alignment [60]. Despite the success of consensus mutations at positions 445 and 446 of BaSP mentioned above, all evaluated full-length consensus enzymes merely displayed an average thermostability, reflecting the diversity of the input sequences used in the design [61].

### 4.2. Structure–Function Relationships in the Active Site

**Figure 4:**
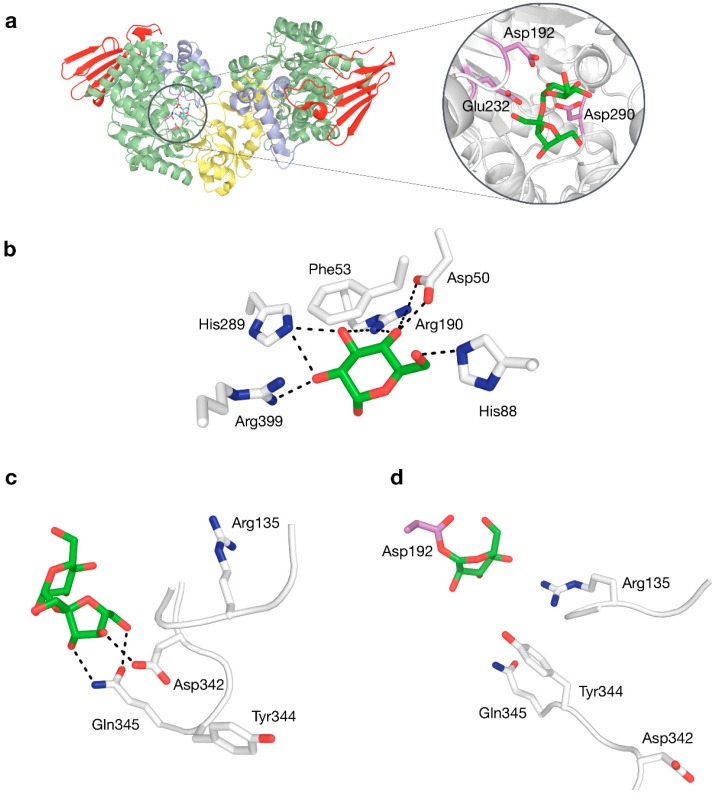
(**a**) Three-dimensional structure of BaSP with a close-up view of sucrose bound to the active site, with the catalytic triad shown in violet. (**b**) Interactions to glucosyl moiety in subsite −1. (**c**) Interactions to fructosyl moiety in subsite +1 by residues in flexible loops. (**d**) Rearranged phosphate-binding conformation of flexible loops in subsite +1.

Structure–function relationships in SP have been studied quite extensively (Table 2). The roles of catalytic nucleophile and acid/base catalyst, both critical in the double displacement mechanism of retaining phosphorylases, are fulfilled by Asp192 and Glu232 in BaSP, respectively. Mutagenesis studies have confirmed that the absence of those side chains eliminates practically all activity [62,63]. A third fully conserved residue that is essential for activity, although not part of the mechanism itself, is the transition state stabiliser Asp290. Its ionised side chain forms a strong hydrogen bond with the 2-hydroxyl group of the glucosyl donor that is considered to promote substrate distortion in the transition state [64]. These three residues thus constitute a crucial catalytic triad, not only in SPs, but in all enzymes in family GH13 (Figure 4a).

Crystal structures of BaSP at different stages of the catalytic cycle are available [9,12]. These have shown that several sidechains in subsite −1 form an extensive hydrogen-bonding network with the glucosyl group of the donor substrate (Figure 4b), explaining why SP is strictly specific for glucosylated donors and why it does not tolerate even minor modifications to the substrate in this binding pocket [65,66,67]. The rigidity of subsite −1 strongly contrasts with the behaviour of subsite +1, which can undergo drastic structural rearrangements, primarily around positions 132–137 and 336–344 [9]. When the subsite switches from its fructose-binding to its phosphate-binding conformation, Asp342 moves out of the binding site, while residues Arg135 and Tyr344 move in (Figure 4c,d). This conformational plasticity is probably partially responsible for the promiscuity of SP.

The contribution of each residue in subsite +1 to the specificity of SP was unequivocally determined by substituting them all to alanine and analysing the influence of those mutations on the affinity for the enzyme’s natural acceptors [70]. The results indicated that Arg135, Leu343 and Tyr344 are important for the affinity for phosphate, while Tyr132 and Asp342 are important for the affinity for fructose. Pro134, Tyr196, His234 and Gln345 contribute to the affinity for both. All findings could be explained by inspecting the structural context of the positions, except for the harsh 3000-fold decrease in phosphorolytic activity when the side chain of His234 is eliminated. The residue is located near the fructosyl group of sucrose but does not seem to interact with the substrate directly, so it remains unclear why its side chain exerts such an essential influence.

Looking at the active site of other enzymes in subfamily GH13_18, there is a clear distinction between subsites −1 and +1. It seems like the former subsite is completely conserved in SP, SPP, GGoP and GGaP, supporting the fact that all of these proteins strictly bind a glucosyl group in that pocket. On the other hand, the positions that correspond to the fructosyl-binding positions of SP are occupied by a different set of residues in enzymes with a different specificity. One can assume that these residues fulfil an important role, especially when they are conserved among all homologs from the same clade, but experimental verification is only available in a few cases (Table 2). In the promiscuous SPP from *T. thermosaccharolyticum*, mutagenesis of Arg134 and His344 causes the activity on fructose 6-phosphate to decrease much more than the activity on fructose, indicating that they may bind the phosphate group of sucrose 6^F^-phosphate [8]. Docking experiments of the substrate in the crystal structure of TtSPP later provided further confirmation of this hypothesis [11]. In the strict SPP from *I. coccineus*, the residues that contribute to the affinity for fructose 6-phosphate, in descending order of importance, are Lys434, Lys364, Arg152 and Tyr377 [11]. Glu264 and Gln378 were predicted to be binding partners too, but their influence has yet to be verified in vitro. Finally, in the GGaP from *M. silvanus* and the GGoP from *M. adhaerens*, a few specificity-determining residues have been uncovered by molecular docking in a homology model followed by mutagenesis [15,18]. Substitution of Asn275 and Glu383 severely decreases the activity of GGaP. In GGoP, Tyr194 and Gln336 both seemingly form a hydrogen bond with a hydroxyl group of glycerol, while Ala333 is appropriately located for hydrophobic interaction with atom C_1′_. They too pronouncedly lower the enzyme’s activity when mutated.

### 4.3. Engineering of Specificity or Selectivity

Despite their great potential for the synthesis of sugars and glycosides with high added value, developing economical production processes with SP and related enzymes remains challenging. As mentioned before, the transglycosylation activity of the promiscuous members on alternative acceptors is rarely significantly higher than the competing hydrolytic activity. Furthermore, they do not always show the desired selectivity. A few engineering efforts have been devoted to overcoming these limitations. Without exception, they were focused only on the +1 subsite.

The first study aimed to alter the preferred regioselectivity of BaSP when presented with glucose as alternative acceptor [71]. BaSP preferentially connects the incoming glucose molecule to the glucosyl moiety of the donor through an α-(1,4) bond, forming maltose. The α-(1,2)-bonded glucobiose kojibiose is generated as a minor product. However, the latter is a non-cariogenic rare sugar with possible prebiotic properties. By fully randomising eleven acceptor site positions, several hits were obtained that notably improved the selectivity and activity towards kojibiose synthesis. All combinations of the hits were characterised too, unveiling that the double mutant L341I-Q345S exhibits a selectivity of 95% with only a modest loss of activity. Variant L341I-Q345S was then used to optimise and scale up an elegant process for the conversion of the bulk sugar sucrose into the rare sugar kojibiose [72]. Starting from 2M sucrose and 200 mM glucose, in the presence of glucose isomerase to convert the side product fructose into glucose, kojibiose concentrations exceeding 1.5 M could be readily achieved with an atom efficiency of 79%. Performing the reaction at a 10-L scale, 3.3 kg of crystalline kojibiose with a purity of over 99.8% could be obtained by simply cooling down the reaction mixture, while an additional 1.2 kg with a purity of 99% could be recovered through crystallisation of the remaining supernatant. This positive result clearly demonstrates both the power and the necessity of enzyme engineering, and it finally enabled a deeper characterisation of the properties of the unfamiliar and expensive sugar in food applications.

**Figure 5:**
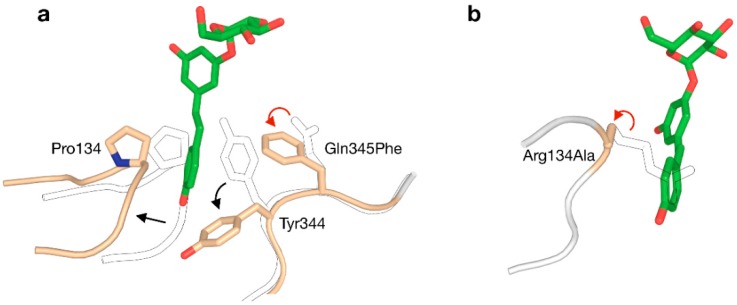
Two strategies for enlarging the acceptor site of GH13_18 phosphorylases to enable glycosylation of resveratrol (red arrow: mutation; black arrow: conformational shift). (**a**) The Q345F mutation in BaSP triggers a clash-induced cascade of conformational changes. (**b**) The R134A mutation in TtSPP broadens the entrance to the active site. The outlined loops and sidechains represent the wild-type.

The +1 subsite of BaSP was also modified in an attempt to promote activity on bulky aromatics like catechin, epicatechin and resveratrol [73]. In a rather unconventional strategy, Gln345 was mutated to Phe with the aim of establishing π-π-stacking-mediated coordination of the acceptor. The resulting variant was indeed a better catalyst for producing glucosides of aromatics, and determination of its crystal structure unveiled that a peculiar clash-induced cascade of loop shifts is responsible for the changed activity (Figure 5a). The larger side chain at position 345 sterically hinders Tyr344, preventing it from interacting with Pro134 in an adjacent loop and subsequently moving the loops apart. Hence, the volume of the active site was doubled and the binding of larger species was facilitated, despite the presence of a bulkier residue in the subsite [74]. A crystal structure of the Q345F mutant later showed that the loop shift is fully reversible, as its sucrose-binding conformation is essentially unaltered [75]. This result again stresses the dynamic nature of the active site of SP.

A somewhat unexpected side effect of the Q345F mutation was that it adjusts the regioselectivity of BaSP when glucose is the acceptor. Rather than producing a mixture of maltose and kojibiose like the wild-type enzyme does, the mutant generates a mixture of maltose and the rare α-(1,3)-bonded nigerose [76]. Curiously, the addition of dimethyl sulfoxide as cosolvent was found to strongly favour the synthesis of nigerose over maltose. In a different study, the Q345F variant was further engineered in order to improve the activity and selectivity of SP towards nigerose formation without the need for a cosolvent. A quadruple mutant (R135Y-D342G-Y344Q-Q345F) was designed that forms nigerose with greater selectivity and a 68-fold improved catalytic efficiency in aqueous solution [77]. Molecular dynamics simulations suggested that the acceptor site of the quadruple mutant is more flexible than the one of the Q345F mutant, offering a possible explanation for its enhanced properties. The quadruple mutant eventually enabled the production of approximately 100 g of nigerose from sucrose and glucose.

Although all above examples were centred around BaSP, other enzymes from GH13_18 can be attractive templates for engineering too. Before the Q345F mutation of BaSP was reported to stimulate the glycosylation of resveratrol, that goal had already been accomplished by redesigning the active site of TtSPP [78]. Despite not being a true SP, TtSPP still shows good affinity for sucrose with the added advantage of already being more active on smaller ‘building blocks’ of resveratrol, i.e., orcinol and resorcinol. A crystal structure of the enzyme had not been published at the time, but a homology model sufficed to predict that the side chain of Arg134 closes the entrance to the active site and limits the size of the binding pocket. By substituting arginine to alanine, a useful affinity for resveratrol was established (Figure 5b). The R134A variant was more promiscuous towards large compounds in general. In a different work, the same enzyme was also used for the challenging glucosylation of 3-hydroxy-β-lactams [79].

Proteins from the clade of strict SPPs, GGoPs and GGaPs have not yet been fully considered as templates for enzyme engineering. Because they are not promiscuous at all, tweaking their substrate preference may appear to be much more complicated. Nevertheless, broadening the acceptor scope of IcSPP was already found to be fairly simple, as various single-point mutations in subsite +1 could implement minor activity on fructose, glycerol and/or glycerate [11].

## 5. Concluding Remarks

The work that has been done with GH13_18 enzymes over the past few years has strengthened their reputation as powerful tools for the synthesis of sugars and glycosides. Thanks to the results of various process and protein engineering efforts, numerous compounds with industrial appeal can now be obtained with the help of SP. However, after some more tinkering with the enzyme’s active site or with the process parameters, it should be possible to expand the list of interesting products of SP even further. Rare or unnatural sugars are perhaps the most valuable targets, because with the global rise of obesity and the rapid growth of the functional foods market, the quest for healthier carbohydrates is now more relevant than ever.

Furthermore, the discovery and characterisation of a few enzymes with novel natural specificities has opened up new directions for the development of useful phosphorylase-mediated biocatalytic processes. Their most exciting application is probably their use as alternative starting points for engineering, as their different active site architectures may happen to be more favourable templates in certain cases. To support such undertakings, it would be helpful to gather more information about their structure–function relationships by determining their crystal structures or by conducting mutational analyses. Moreover, it may be worth trying to introduce significant activity on sucrose in the enzymes with a more stringent specificity. Doing so would diversify the repertoire of catalysts that can be applied to generate valuable glycosidic products from this cheap bulk sugar.

Finally, plenty of questions remain to be answered about the natural function of the newly discovered GH13_18 enzymes. GGoP and GGaP were missing pieces of the metabolic puzzle of compatible solutes, but their exact purpose in vivo has yet to be investigated more deeply. In addition, it would certainly be interesting to study the mysterious myxobacterial phosphorylases further, considering how their source organisms are rich sources of unique biosynthetic routes and metabolites. Subfamily GH13_18 clearly not only houses enzymes with promising biotechnological potential but also enzymes that can provide more insight into poorly understood microbial pathways.

## Figures and Tables

**Table 1:** One-pot enzyme cascade reactions that include SP activity for the generation of Glc1P. Yields are relative to sucrose.

Product	Enzymes	Substrates and Cofactors	Reaction Conditions	Yield	Ref.
β(1,4)-Galactosyl l-rhamnose	*B. longum* SP, UDP-glucose—hexose 1-phosphate uridylyltransferase, UDP-glucose 4-epimerase, Galactosyl l-rhamnose phosphorylase	1.1 M sucrose, 1 M l-rhamnose, 30 mM phosphate, 1 mM UDP glucose, 10 mM MgCl_2_	30 °C, pH 7	65%	[35]
β(1,4)-Mannosyl N-acetylglucosamine	SP (unknown source), α-phosphoglucomutase, glucose 6-phosphate isomerase, mannose 6-phosphate isomerase, α-phosphomannomutase, β(1,4)-mannosyl N-acetylglucosamine phosphorylase	250 mM sucrose, 250 mM N-acetylglucosamine, 25 mM phosphate, 60 µM glucose 1,6-bisphosphate	30 °C, pH 7	23%	[36]
Nigerose	*B. longum* SP, nigerose phosphorylase, xylose isomerase, α-phosphoglucomutase, β-phosphoglucomutase	500 mM sucrose, 25 mM phosphate, 10 mM MgCl_2_, 41 µM glucose 1,6-bisphosphate	30 °C, pH 7	67%	[37]
Amylose	*T. thermosaccharolyticum* SPP, potato α-glucan phosphorylase	100 mM sucrose, 50 mM phosphate, 200 µM maltodextrins	37 °C, pH 7.4	0.22 g/g ^1^	[33]
Short-chain cellodextrins	*B. adolescentis* SP, cellobiose phosphorylase, cellodextrin phosphorylase	200 mM sucrose, 50 mM phosphate, 65–80 mM glucose	45 °C, pH 7	~90%	[34]
Glucaric acid	*L. mesenteroides* SP, phosphoglucomutase, myo-inositol 1-phosphate synthase, myo-inositol monophosphatase, myo-inositol oxygenase, uronate dehydrogenase, NADH oxidase	50 mM sucrose, 2 mM MgCl_2_, 2 mM Fe^2+^, 3 mM NAD^+^	30 °C, pH 7.5	75% ^2^	[38]

^1^ Gram product per gram of sucrose. ^2^ With fed-batch addition of NaOH and myo-inositol oxygenase.

**Table 2:** Function of residues in GH13_18 phosphorylases. Residue numbers are from the sequence of *B. adolescentis* SP (also for catalytic residues), *T. thermosaccharolyticum* promiscuous SPP, *I. coccineus* strict SPP, *M. adhaerens* GGoP or *M. silvanus* GGaP. When no reference is given, the potential function was derived from the crystal structure of BaSP (PDB code 2GDU).

Enzyme	Residue	(Potential) Function	Ref.
All	Asp192	Catalytic nucleophile	[63]
	Glu232	Catalytic acid/base	[62]
	Asp290	Transition state stabiliser	[64]
	Phe53	Hydrophobic platform, transition state stabilisation, stacking interaction in subsite −1	[68]
SP	Asp50	Binding of glucosyl moiety (H-bond with OH4)	[69]
	His88	Binding of glucosyl moiety (H-bond with OH6)	-
	Arg190	Binding of glucosyl moiety (H-bond with OH2)	-
	His289	Binding of glucosyl moiety (H-bond with OH2 and OH3)	-
	Arg399	Binding of glucosyl moiety (H-bond with OH3 and OH4)	[69]
	Tyr132	Indirectly involved in binding of fructosyl moiety (via Tyr196)	[70]
	Pro134	Indirectly involved in binding of phosphate and fructosyl moiety	[70]
	Arg135	Binding of phosphate	[70]
	Tyr196	Binding of fructosyl moiety (hydrophobic interaction with C1) and indirectly influences phosphate binding (via Tyr344)	[70]
	His234	Crucial for overall activity (reason unknown)	[70]
	Asp342	Binding of fructosyl moiety (H-bond with OH4)	[70]
	Leu343	Indirectly involved in binding of phosphate (via Tyr344)	[70]
	Tyr344	Binding of phosphate	[70]
	Gln345	Binding of fructosyl moiety (H-bond with OH3 and OH6) and indirectly influences phosphate binding (via Tyr344)	[70]
SPP	Arg134	Binding of the phosphate group of fructose 6-phosphate	[8]
(promiscuous)	His344	Binding of the phosphate group of fructose 6-phosphate	[8]
SPP	Arg152	Binding of fructose 6-phosphate	[11]
(strict)	Lys364	Binding of fructose 6-phosphate	[11]
	Lys434	Binding of fructose 6-phosphate	[11]
GGoP	Tyr194	Binding of glycerol	[18]
	Ala333	Binding of glycerol	[18]
	Gln336	Binding of glycerol	[18]
GGaP	Asn275	Binding of glycerate	[15]
	Glu383	Binding of glycerate	[15]

## Supplementary Materials

Supplementary materials can be found at https://www.mdpi.com/1422-0067/21/7/2526/s1.

## Abbreviations

BaSP	*B. adolescentis* SP
CAZy	Carbohydrate-active enzyme database
GGaP	2-*O*-Glucosylglycerate phosphorylase
GGoP	2-*O*-Glucosylglycerol phosphorylase
GH13_18	Glycoside hydrolase family 13, subfamily 18
Glc1P	α-d-Glucose 1-phosphate
iCLEA	Imprinted cross-linked enzyme aggregate
IcSPP	*I. coccineus* SPP
LmSP	*L. mesenteroides* SP
SP	Sucrose phosphorylase
SPP	Sucrose 6^F^-phosphate phosphorylase
TtSPP	*T. thermosaccharolyticum* SPP
