# Supplementary material for: Sucrose Phosphorylase and Related Enzymes in Glycoside Hydrolase Family 13: Discovery, Application and Engineering
Source: Int J Mol Sci. 2020 Apr 5;21(7):2526. doi: 10.3390/ijms21072526 (PMC7178133; doi:10.3390/ijms21072526)
Supplement: Supplementary file 1 [file ijms-21-02526-s001.pdf]

Supplementary Information for

Sucrose phosphorylase and related enzymes in glycoside hydrolase family 13: discovery, application and engineering

International Journal of Molecular Sciences

Jorick Franceus and Tom Desmet

Corresponding author:
Tom Desmet, Centre for Synthetic Biology
Email: tom.desmet@ugent.be
Website: www.biocatalysis.ugent.be

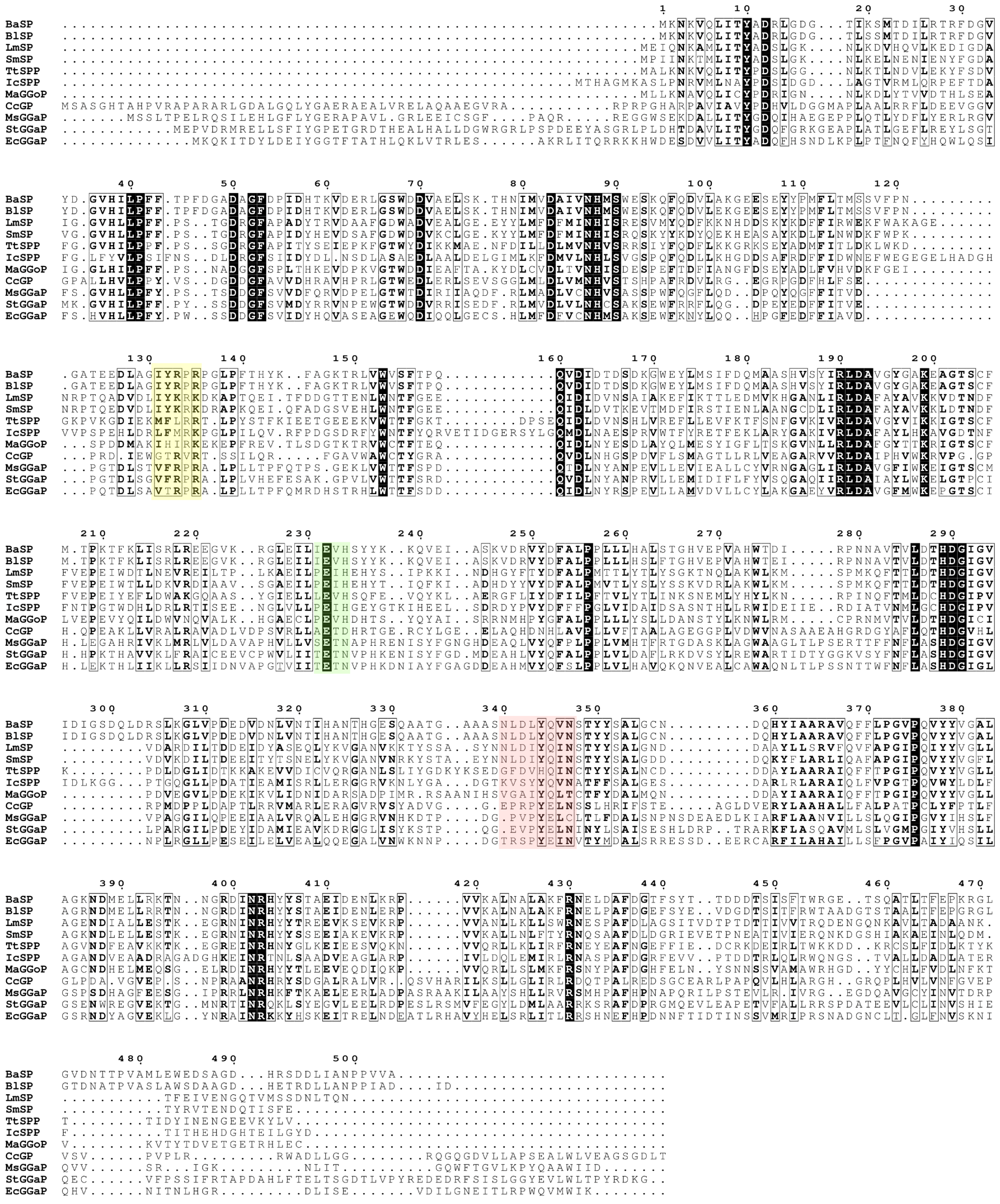

Figure S1 Multiple sequence alignment of characterised enzymes in subfamily GH13_18 (BaSP: *Bifidobacterium adolescentis* SP; BlSP: *B. longum* SP; LmSP: *Leuconostoc mesenteroides* SP; SmSP: *Streptococcus mutans* SP; TtSPP: *Thermoanaerobacterium thermosaccharolyticum* SPP; IcSPP: *Ilumatobacter coccineus* SPP; MaGGoP: *Marinobacter adhaerens* GGoP; CcGP: *Corallococcus coralloides* glycoside phosphorylase; MsGGaP: *Meiothermus silvanus* GGaP; StGGaP: *Spirochaeta thermophila* GGaP; EcGGaP: *Escherichia coli* GGaP). The alignment was visualised using ESPript 3.0 (http://espript.ibcp.fr). Important acceptor site loops were highlighted in yellow (loop B), green (catalytic acid/base residue loop) and red (loop A).
